# Preferential Solvation by Trifluoroethanol Drives *α*‐Helical Folding in the Disordered S2 Region of the Escargot Protein

**DOI:** 10.1002/cphc.202500668

**Published:** 2026-02-10

**Authors:** Vinicius Piccoli, Ander F. Pereira, Lina Rivillas‐Acevedo, Nina Pastor, Ángel E. Peláez‐Aguilar, Leandro Martínez

**Affiliations:** ^1^ Universidade Estadual de Campinas (UNICAMP) Institute of Chemistry and Center for Computing in Engineering & Science Campinas São Paulo Brazil; ^2^ Universidad Autónoma del Estado de Morelos Centro de Investigación en Dinámica Celular Cuernavaca Morelos Mexico; ^3^ Departamento de Microbiología Molecular Universidad Nacional Autónoma de México Instituto de Biotecnología Cuernavaca Morelos Mexico

**Keywords:** 2,2,2‐trifluoroethanol, helices, protein folding, solvation

## Abstract

The N‐terminal domain of the *Drosophila* *melanogaster* Escargot transcription factor (Esg) is an intrinsically disordered region (IDR) that complements the DNA‐binding activity of its C‐terminal zinc fingers. Within this IDR, the S2 segment (residues 120–152) is predicted to form an *α*‐helical molecular recognition feature, a transient structural element implicated in protein–protein interactions. We examined the conformational equilibrium of the S2 peptide in water and in helix‐promoting 2,2,2‐trifluoroethanol (TFE)/water solutions using replica exchange with solute tempering 2 (REST2) simulations and circular dichroism measurements. We show that the peptide can display substantial ellipticity, with TFE nearly doubling the helix population at 40% v/v compared to pure water. Minimum‐distance distribution functions and the Kirkwood–Buff theory of solvation show that TFE preferentially accumulates on the peptide domain. This effect primarily arises from nonspecific contacts between TFE and uncharged polar and nonpolar side chains of the peptide. These findings support the view that the S2 region's structural plasticity is critical for modulating the function of Esg and provide further insights into TFE‐induced helix stabilization.

## Graphical abstract

A disordered S2 region of the Escargot protein forms helices which are stabilized by TFE and are correlated with molecular recognition.

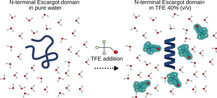

## 1 Introduction

The Escargot (Esg) protein from *Drosophila melanogaster* is a transcription factor from the S6nail family. The gene coding Esg is highly pleiotropic and plays essential, diverse roles across a wide developmental window in Drosophila melanogaster [1]. It is a critical regulator of stemness and pluripotency, suppressing differentiation in intestinal stem cells [2, 3], inhibiting neuroblast differentiation in the nervous system [4], and maintaining stem cell populations in the testes [5]. Its functions also extend to regulating cell ploidy, longevity, and metabolism [6]. This functional multiplicity across disparate tissues, such as the gut, imaginal discs, and neuroblasts, suggests that Esg must interact with a diverse set of molecular partners. Such functional adaptability is characteristic of proteins containing intrinsically disordered regions and molecular recognition features (MoRFs), as these flexible domains are uniquely suited to mediate numerous specific, yet transient, protein–protein interactions [7].

The Esg protein is characterized by a structured C‐terminal domain with five zinc fingers and a large N‐terminal domain. While the C‐terminal domain is responsible for DNA binding [8, 9], the N‐terminal domain is involved in complementary functions, including protein degradation [10, 11], but it is expected to be predominantly disordered [12]. Nevertheless, a 45‐amino‐acid segment within this domain, termed the S2 region (residues 120–152), was predicted to form an *α*‐helical MoRF (*α*‐MoRF) [13].

The investigation of the structural properties of the N‐terminal domain of Esg is crucial for dissecting its possible function. For instance, the Esg protein contains a Pro‐X‐Asp‐Leu‐Ser‐X‐Lys (P‐DLS‐K) [14] domain that allows it to interact with the *Drosophila* C‐terminal binding protein (dCtBP) corepressor independently of the C‐terminal DNA‐binding zinc fingers [4, 12]. Importantly, Esg is the *Drosophila* ortholog of the vertebrate Snai1 transcription factor, a master regulator of the epithelial‐to‐mesenchymal transition process, which is crucial in embryonic development and progression of diseases [15, 16, 17]. Therefore, obtaining insights into Esg's function by structural analysis of its disordered regions can provide critical insights into mechanisms of cell plasticity.

To provide insights into possible structure–function relationships of the N‐terminal domain of Esg, we investigate here the hypothesis that the S2 peptide can form transient helical motifs. The helical propensity of the peptide is studied in water and in aqueous solutions of 2,2,2‐trifluoroethanol (TFE), an osmolyte known to stabilize helical folds [18, 19]. The identification of stable helical conformations in TFE is an indication that transient folds can exist in water and play significant roles in the molecular recognition and function of the S2 fragment.

Additionally, from a fundamental thermodynamics perspective, the detailed study of TFE–protein interaction by molecular simulations can provide additional insights into the role of this osmolyte on the protein folding equilibrium [20, 21, 22, 23]. Cosolvents are frequently employed in experimental and computational studies to investigate protein stability and folding pathways. TFE, in particular, has been shown to interact directly with the side chains and backbones of helical peptides. The interactions with the backbones are demonstrated to destabilize the helices [24], such that either direct interactions with the side chains or indirect effects on the solvent structure are responsible for TFE‐induced helix stabilization [25]. Understanding these interactions at a molecular level, particularly when involving disordered domains, is challenging, requiring specialized methods for solvent structure analysis.

In this work, we investigate the influence of TFE on the conformational stability of the S2 peptide from the Esg protein. We synthesized the peptide and measured its secondary structure with circular dichroism (CD) spectroscopy in TFE/water solutions. Using extensive MD simulations with the replica exchange with solute tempering 2 (REST2) enhanced sampling method [26, 27, 28], the use of minimum‐distance distribution functions (MDDFs), and the Kirkwood–Buff theory of solvation, we characterize the conformational ensemble of the peptide in both pure water and TFE/water solutions. By analyzing the structural changes in the peptide and the corresponding reorganization of the solvent, we demonstrate that the S2 peptide can effectively form helical structures, and we elucidate the molecular mechanism by which TFE modulates the stability of the native *α*‐MoRF identified in this critical region of the Esg protein.

## 2 Methods

### 2.1 Structure

Here, we study the effect of TFE in the conformational stability of the S2 region of the Esg protein (sequence: ^120^VPTPTYPKYPWNNFHMSPYTAEFYRTINQQGHQ^152^). The initial structure was modeled using the I‐Tasser predictor [29, 30] with charged N‐terminal and C‐terminal groups.

### 2.2 Enhanced Sampling in Molecular Dynamics Simulations

We employed the REST2 method [28] to sample peptide conformations. In this method, all replicas are simulated at the same temperature, but the intraprotein (*E*_pp_) and protein–solvent (*E*_ps_) interaction potentials are scaled such that the final energy of a conformation *X* is given by

(1) $$E_{m}^{\mathrm{REST}}(X)=\frac{\beta_{m}}{\beta_{0}}E_{\mathrm{pp}}(X)+\sqrt{\frac{\beta_{m}}{\beta_{0}}E_{\mathrm{ps}}(X)}+E_{\mathrm{ss}}(X)$$

where *E*_ss_ is the solvent–solvent interaction energy and *β*_m_ < *β*_0_. Here, *β*_m_ and *β*_0_ correspond to 1/*k*_B_ *T*_m_ and 1/*k*_B_ *T*_0_, respectively. *β*_0_ is defined as the inverse of the base temperature *T*_0_, which corresponds to the physical temperature of the coldest replica in the replica exchange simulation [28]. In contrast, *β*_m_ represents the inverse of an effective temperature *T*_m_ associated with each replica labeled m. Aside from replica 0, every replica is assigned a distinct effective temperature such that *T*_0_ < *T*_1_ <  *T*_2_ < … < *T*_max_. Consequently, the *β* values follow the sequence *β*_0_ > *β*_1_ > *β*_2_ > … > *β*_max_. Note that here, the replica temperature scale is used only to weight the interaction potentials, and not as effective temperatures of the replicas.

In Equation (1), *β*_m_ and *β*_0_ combine to define a scaling factor that modifies the potential energy surface of each replica. Because *β* is inversely proportional to temperature, the ratio *β*_m_/*β*_0_ becomes smaller than 1 for replicas where greater temperatures were used to calculate the scaling factor. This scaling factor is then used to attenuate the solute‐related energy terms (*E*_pp_ and *E*_ps_). In this framework, *β*_0_ defines the true physical temperature of the simulation, while the set of *β*_m_ values generates a series of progressively “softened” potential energy landscapes for the solute, facilitating enhanced sampling across replicas [28].

The REST2 implemented in GROMACS (v2019.4) [31] patched with PLUMED (v2.5.5) [32] was used. All systems were simulated at 300 K, and the interaction potentials *E*_pp_ and *E*_ps_ were scaled by a *β*_m_/*β*_0_ and √(*β*_m_/*β*_0_), respectively, with *β*_m_/*β*_0_ varying from 1 to 0.71. The number of replicas chosen for each system was 10. With this, the acceptance ratio was around 40%.

All systems were initially minimized by 1000 steepest descent steps, followed by two successive 1 ns simulations of equilibration using the Canonical (NVT) and Isotermic‐Isobaric (NPT) ensembles. After the equilibration steps, each replica was simulated on the NPT ensemble for 500 ns (totaling 5 μs for each system, considering the replicas), with exchange attempts every 400 MD steps. Structures were saved every 500 ps. A pressure of 1 bar was used, controlled by the Parrinello–Rahman [33] thermostat with a relaxation time of 2 ps and isothermal compressibility of 4.5 × 10^−5^ bar^−1^. A stochastic velocity‐rescaling thermostat was used to control the temperature with a relaxation time of 0.1 ps [34]. Periodic boundary conditions were applied. A cutoff of 1.2 nm was used for short‐range interactions. Long‐range electrostatic interactions were calculated with the particle–mesh Ewald [35] summation method with a fourth‐order interpolation and a grid spacing of 0.16 nm. All bonds involving hydrogen atoms were constrained with the LINCS algorithm [36]. The leap‐frog algorithm with a time step of 2 fs was used to integrate the equations of motion. For analyses, only the trajectories of the unscaled potentials (*β*_m_/*β*_0_ = 1) were used.

**FIGURE 1:**
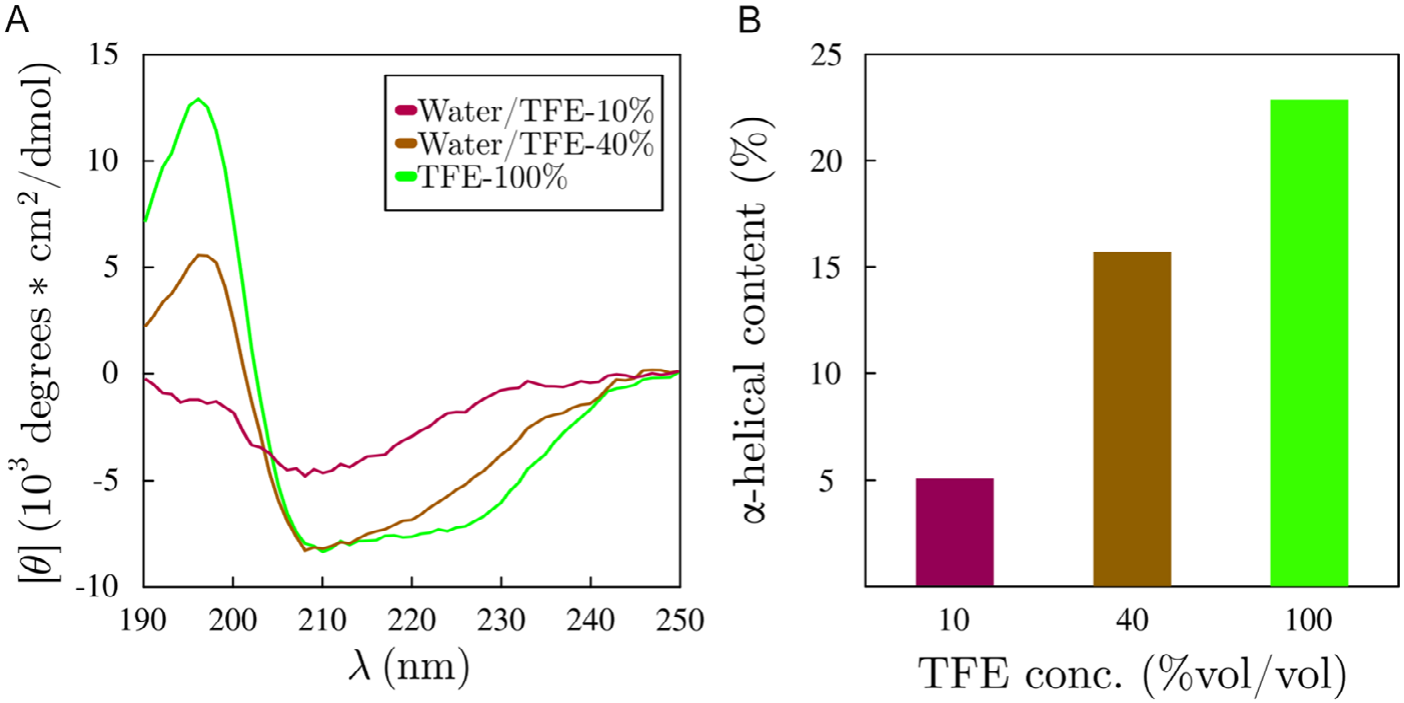
Secondary structure of the S2 region of the Esg protein. (A) The circular dichroism spectra at different concentrations of TFE. (B) The helical content at different concentrations of TFE, estimated by the deconvolution of the spectra in the BeStSel server.

**FIGURE 2:**
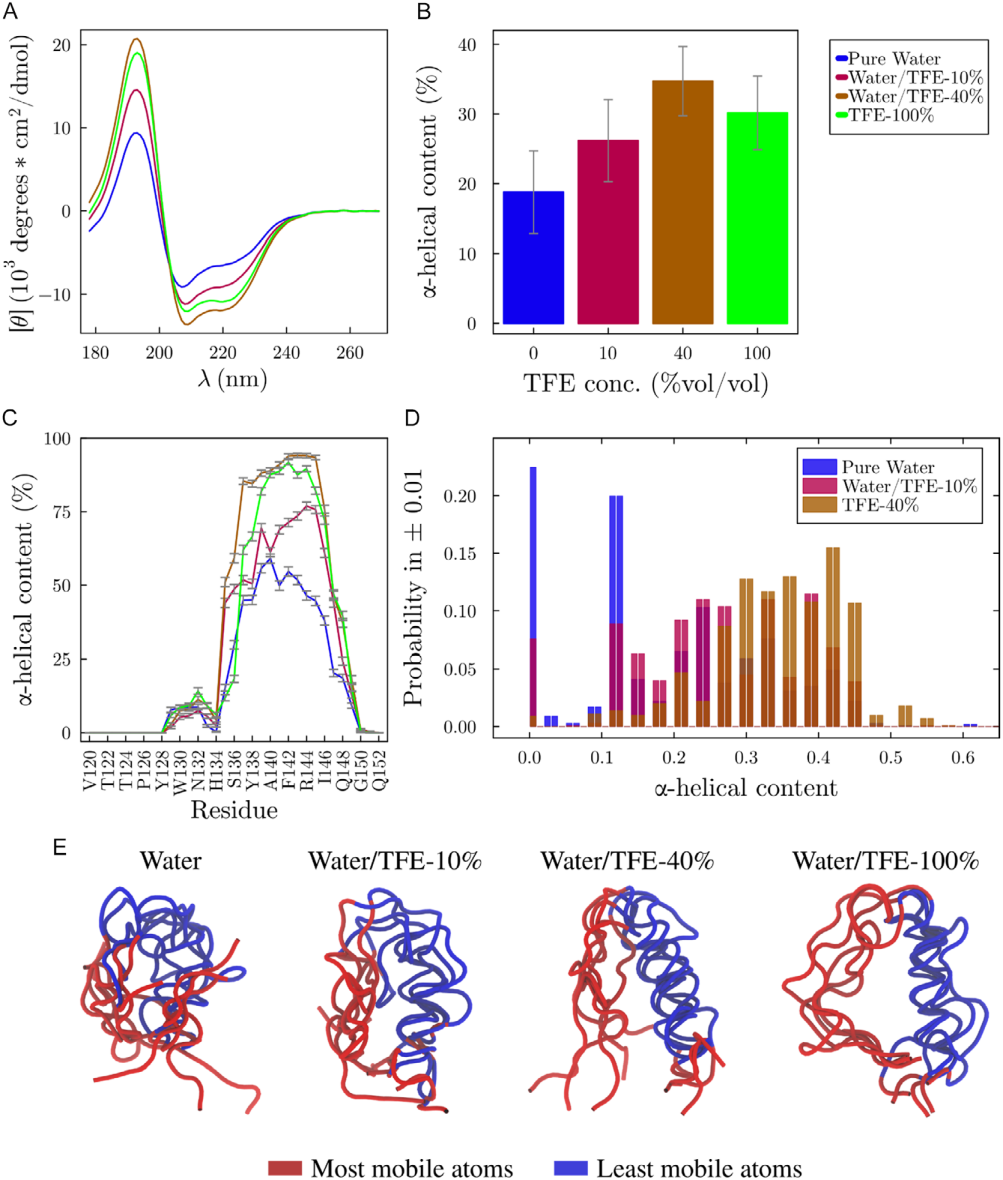
Structural properties of the S2 region of the Esg protein in water (blue) and aqueous solutions of TFE. (A) The increase in peaks at ~190 nm and a double dip at 200–230 nm in circular dichroism (CD) spectra indicates an increase in the helical content of the protein in TFE solutions. (B) Average ellipticity of the peptide. (C) Average per‐residue α‐helix prevalence. (D) Distribution of the ellipticity of the peptide in water and TFE solutions. The error bars in (B) and (D) indicate the standard error of the mean of the quantities computed. (E) Representative structures of the ensembles, aligned to highlight preserved structural regions [43].

The initial configuration of the S2 region of the Esg protein was solvated in cubic boxes of 84 Å with Packmol [37, 38], containing different concentrations of water and TFE (Table 1). One chloride ion was added to the boxes to neutralize the total charge. In this work, we used the TIP4P/2005 water model [39] and the Amber03w force field [40] for the protein. The model used to describe cosolvent molecules (TFE) was developed to reproduce the thermodynamic properties of aqueous solutions of TFE [41]. Best and Mittal showed that the amber03w variant, used in combination with the TIP4P/2005 water model, reproduces experimental helical content of short peptides reasonably well, albeit with a slight overestimation [40]. In this work, we adopted force fields shown to be mutually compatible (amber03w for the peptide, TIP4P/2005 for water, and the TFE model from Vymětal et al.) [40, 41, 42]. Importantly, Vymětal and coworkers demonstrated that this combination is compatible and reliable for simulations of peptides in TFE/water mixtures [41, 42]. While this combination tends to overestimate helicity, we note that the simulations (Figure 2) remain in qualitative agreement with the experimental CD data (Figure 1).

**TABLE 1:** Compositions of molecular systems simulated at different concentrations of 2,2,2‐trifluoroethanol (TFE).

Systems	Number of water molecules	Number of TFE molecules
Escargot – 0% v/v	19,599	0
Escargot – 10% v/v	16,854	494
Escargot – 40% v/v	12,094	1953
Escargot – 100% v/v	0	4890

Structural alignment (Figure 2E) was performed with MDLovoFit [43, 44], as implemented in MolSimToolkit.jl, to highlight preserved vs. mobile regions.

### 2.3 Calculation of Protein Ellipticity and Convergence of the Simulations

The protein ellipticity for each system was estimated both by (a) secondary structure assignment with the DSSP software [45] and by (b) theoretical CD spectra using the SESCA [46] and the HBSS‐3SC1 basis set [24]. We used block averaging, as implemented in the MolSimToolkit.jl package, to assess the convergence of the simulations. The results of standard errors of the estimates and the autocorrelation functions of the average protein helical content in all simulations are available in the Supporting Information (Section 2).

### 2.4 Analysis of the Protein Solvation

This section outlines the theoretical formalism and computational methods used to determine MDDFs and the Kirkwood–Buff integrals (KBIs). All calculations were performed using the ComplexMixtures.jl package [47], and comprehensive theoretical details can be found in previous publications [48, 49, 50].

The system under investigation is a tertiary solution containing a peptide (species p), water (species w), and the TFE cosolute (species c). The peptide is treated as being at infinite dilution within the water–cosolvent mixture, and the molar concentrations of water and cosolvent are, respectively, *ρ*_w_ and *ρ*_c_. The cosolvent distribution around the peptide in the solution can be described in terms of the average number density of cosolvents *n*_c_(*r*) relative to the density of an ideal‐gas distribution, $n_{c}^{*}(r)$:

(2) $$g_{\mathrm{pc}}(r)=\frac{n_{c}(r)}{n_{c}^{*}(r)}$$

where *r* is the minimum distance between any peptide and cosolvent atoms and *g*_pc_(*r*) is the MDDF [51, 52]. The MDDFs provide a detailed analysis of the molecular interactions and can be used to calculate thermodynamic quantities through the Kirkwood–Buff theory of solutions.

The KBIs can be calculated from *n*_c_(*r*) and $n_{c}^{*}(r)$ by

(3) $$G_{\mathrm{pc}}=\frac{1}{\rho_{c}}\int_{0}^{\infty }[n_{c}(r)-n_{c}^{*}(r)]S(r)\;dr$$

where *S*(*r*) is the area defined by the minimum distance *r* to the solute and is dependent on the solute's shape. Using Equation (3), we obtain, for a large‐enough finite subvolume of the system,

(4) $$G_{\mathrm{pc}}(R)\approx \frac{1}{\rho_{c}}[N_{\mathrm{pc}}(R)-N_{\mathrm{pc}}^{*}(R)]$$

where *N*_pc_(*R*) and $N_{\mathrm{pc}}^{*}(R)$ are, respectively, the number of minimum distances between the protein and the solvent smaller than *R* and the number of equivalent distances within *R* in a system with ideal‐gas distribution (i.e., in the absence of solute–solvent interactions) [48, 49, 50]. Here, *R* is a distance large enough to encompass the region of the solution where solvent molecules display significant correlations with the solute (the peptide). This region is known as the “protein domain.” In practice, it will be the maximum distance at which the MDDFs and KBIs will be computed, and the lack of correlation at *R* can be inferred by the convergence of distribution functions and KBIs.

The preferential solvation parameter is a thermodynamic quantity that can be experimentally determined from, for example, equilibrium dialysis and vapor pressure osmometry [53, 54]. It can be calculated from the difference of the KB integrals of the solvent components [55, 56, 57]. When the solute is considered infinitely diluted, the preferential binding of the cosolvent to the protein, relative to water, is

(5) $$\Gamma_{\mathrm{pc}}(R)\approx \rho_{c}[G_{\mathrm{pc}}(R)-G_{\mathrm{pw}}(R)]$$

and consists of the number of cosolvent molecules in excess or deficit in the protein domain, considering the cosolvent molecular volume in the bulk solution. The binding of water relative to the cosolvent can be provided by the preferential hydration parameter:

(6) $$\Gamma_{\mathrm{pw}}(R)\approx \rho_{w}[G_{\mathrm{pw}}(R)-G_{\mathrm{pc}}(R)]$$

A positive Γ_pc_(*R*) (and negative Γ_pw_(*R*)) means that the cosolvent accumulates in the protein domain, such that the protein is effectively dehydrated.

The MDDFs were calculated using a discretized version of Equation (2) in which the density was computed from the average number of minimum distances at each 0.1 Å bin. The KBIs and preferential interaction parameters for the cosolvent were calculated according to Equations (4) and (5) and the preferential hydration parameter according to Equation (6).

The effective bulk concentrations for each solvent were computed within an open subdomain of the simulation box chosen to minimize finite‐size effects. This was achieved by calculating the solvent densities for molecules within 10–15 Å from the solute's surface [58]. The selection of this distance range is supported by the fact that MDDFs and the KB integrals converged for radii *R* ≥ 10 Å across most systems studied.

### 2.5 Peptide Synthesis and Purification

The peptide VPTPTYPKYPWNNFHMSPYTAEFYRTINQQGHQ was synthesized by solid‐phase synthesis using the Fmoc strategy and Rink amide resin, as previously described [59, 60]. The peptide was acetylated at the amino terminus and amidated at the carboxylic terminal. Its molecular weight was determined by electrospray ionization mass spectrometry, and it was purified by semipreparative reverse‐phase C18 high‐performance liquid chromatography.

### 2.6 CD Spectroscopy

CD spectra were run at room temperature in the UV region, from 190 to 250 nm, using a Jasco J‐815 spectropolarimeter. Spectra were recorded in quartz cells with 0.1 cm path lengths. The peptide was diluted in TFE to a final concentration of 10 μM, and the TFE concentration varied from 10% to 100%. The secondary structure analysis was made with the BeStSel software [61, 62].

## 3 Results and Discussion

### 3.1 Structural Properties of the S2 Region of the N‐Terminal Domain of the Esg Protein

The C‐terminal zinc fingers of the Esg protein are conserved and well‐known for their role in DNA recognition and transcription factor [1, 63]. In contrast, the functions of the N‐terminal domain remain less understood. This region is intrinsically disordered, although a sequence of 33 residues (residues 120−152, here referred to as the S2 region) displays a propensity to adopt an *α*‐helical structure [13]. Trifluoroethanol (TFE), a well‐known helix‐inducing agent, can be used to probe which segments of the N‐terminal domain exhibit higher helical propensity [24, 64]. Understanding the structural features of this disordered region may provide insight into its potential role in ligand recognition and function that complements the DNA‐binding activity of the Esg protein [13, 63].

Figure 1A shows the experimental CD spectra of the protein in three TFE solutions with increasing concentration. It should be noted that this peptide is hydrophobic and requires DMSO to be dissolved in water; as DMSO interferes with CD measurements, the ellipticity without TFE could not be measured. Figure 1B shows the deconvolution of the CD spectra with the BeStSel server, demonstrating an increase in helical content as the TFE concentration increases.

Figure 2A shows the theoretical CD spectra of the protein in water (blue) and in TFE solutions, computed from the molecular dynamics simulations. All spectra in Figures 1 and 2 are similar to those expected for proteins with *α*‐helix content, i.e., containing a positive band around 193 nm and two other negative bands at 208 and 222 nm [65]. As indicated previously, the S2 region of the Esg protein forms *α*‐helices [13]. In the presence of TFE, all peaks become more pronounced, highlighting the stabilizing role of the cosolvent on the secondary structure. The experimental and simulated helical contents agree qualitatively concerning the effect of TFE, but the simulations appear to overestimate the content at lower TFE concentration, with the TFE helical stabilization peaking at ∼40%. This overestimation is expected from the force‐field combination used, as discussed in Section 2.2 [40].

Figure 2B confirms that the total *α*‐helical content of the protein in TFE is considerably higher than in pure water, even in the systems where the cosolvent concentration is low (10% v/v). Note that at the concentration of 40% TFE, where the simulated stabilizing effect of TFE is maximum, the *α*‐helix content is almost twice as high as in pure water. The stabilizing role of TFE does not occur for the entire S2 region of the Esg protein. According to Figure 2C, the average per‐residue *α*‐helical content shows that the N‐terminal (V120‐Y128) and C‐terminal (G150‐Q153) regions are disordered in all solutions. Notably, three (among nine) residues of the N‐terminal region are proline, explaining the lack of structure of this region [66, 67]. In contrast, the central segment (M135‐Q149), which was identified as having high *α*‐helical propensity [13], is significantly stabilized in TFE solutions. Interestingly, a short segment (P129–F133) exhibits a small but consistent *α*‐helical content across all conditions, indicating that it remains relatively unaffected by changes in cosolvent concentration.

Figure 2D shows that, in all simulations, the peptide samples conformations with both high and low *α*‐helical contents. The population of the coiled state in water is maximal and minimal in the 40% vol/vol TFE solution. Representative ensembles of aligned structures from the simulations are shown in Figure 2E. In water, the structures appear more globular yet largely unstructured. On the other hand, in 40% TFE, the N‐ and C‐terminal regions remain disordered, while the central segment is predominantly helical. This structural behavior is further supported by the solvent‐accessible surface area shown in Table S1, which are lowest in water and progressively increase with TFE concentration, indicating a shift toward more solvent‐exposed but structurally ordered conformations.

### 3.2 MDDFs

**FIGURE 3:**
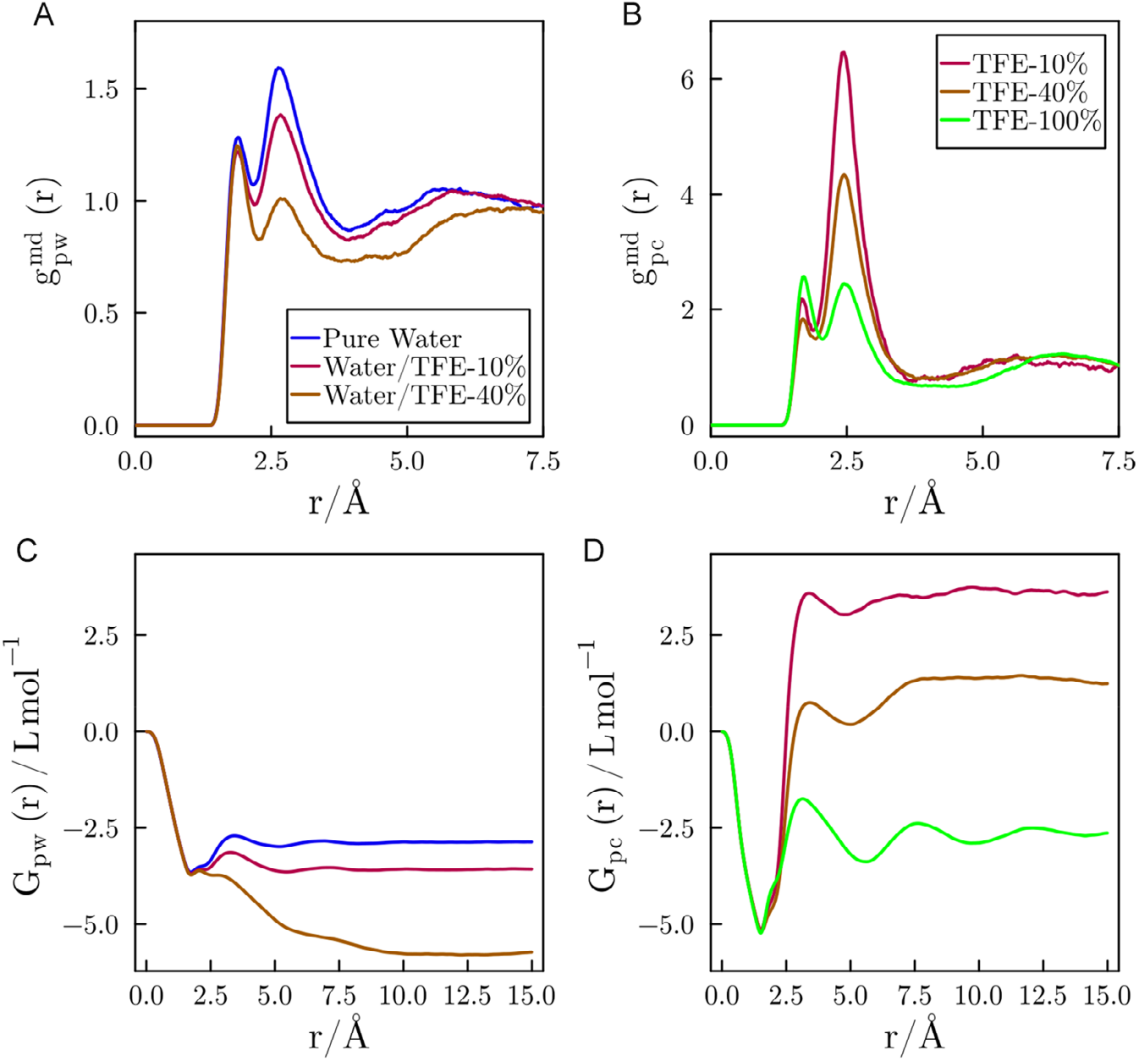
Minimum‐distance distribution functions of (A) water and (B) TFE as a function of cosolvent concentration, including the pure water (blue) and pure TFE (100%, green) conditions. Panels (C) and (D) show the corresponding KBIs for water and TFE, respectively.

Figure 3A,B depicts the MDDFs for water and TFE in systems containing pure water and 10%, 40%, and 100% (v/v) TFE solution. Both water and TFE display peaks centered at ∼1.8 Å. These peaks are due to specific interactions, i.e., hydrogen bonds, while the second peaks, at ∼2.6 Å, are characteristic of the second solvation shell and nonspecific interactions. In the presence of TFE, the relative density of water in the second solvation shell notably decreases with increasing cosolvent concentration, while the first peak remains almost unaltered. The results indicate that the affinity of water at hydrogen‐bonding distances with the protein is not affected by the presence of TFE. At the same time, since the water concentration at bulk is smaller, this means that the effective number of hydrogen bonds of water with the peptide decreases with increasing concentration of TFE. TFE also forms hydrogen bonds with the protein, but with a much lower proportion than nonspecific interactions. As previously reported, direct interactions of TFE with the protein are destabilizing for the helices [24]. Interestingly, the peak at hydrogen‐bonding distances for TFE is minimal at 40% vol/vol TFE, where the protein has the maximum *α*‐helix content.

Figure 3C,D presents the KBIs for water and TFE, which measure the excess accumulation or deficit of each solvent around the peptide. For both solvents, the integrals show a sharp initial drop at short distances (*r* < 1.5 Å), a feature corresponding to the excluded volume of the peptide and solvent molecules. Beyond this region, favorable solute–solvent interactions can lead to a positive slope, indicating solvent accumulation.

The KBI for water (Figure 3C) shows that in pure water and in 10% TFE solutions, there is only a slight recovery after the initial volume exclusion. At 40% TFE, however, the integral becomes significantly more negative, demonstrating that water is largely displaced from the peptide surface. This suggests that the presence of TFE weakens peptide–water affinity. Conversely, the KBI for TFE (Figure 3D) shows a net accumulation at both 10% and 40% (v/v). Notably, the accumulation is less pronounced at 40% TFE, suggesting that while TFE is still preferred over water, its effective affinity for the peptide decreases as its own bulk concentration increases. For comparison, the KBI in pure TFE (100% v/v) is negative, confirming that the strong preferential solvation of the peptide by TFE is a synergistic effect specific to the aqueous mixture.

These findings are quantitatively summarized by the preferential interaction parameters (Γ_pc_), calculated using Equation (5). As shown in Table 2, Γ_pc_ is positive for both 10% and 40% TFE solutions, confirming that TFE preferentially solvates the protein in these mixtures.

**TABLE 2:** Effective concentrations of TFE solutions, Kirkwood–Buff integrals for water (*G*_pw_) and TFE (*G*_pc_) relative to the S2 region of the Esg protein, and preferential parameter solvation (Γ_pc_).

Systems	Water concentrations, mol L^−1^	TFE concentrations, mol L^−1^	*G*_pw_, L mol^−1^	*G*_pc_, L mol^−1^	Γ_pc_
Esg – TFE 10%	50.12	1.43	−3.58	3.61	10.29
Esg – TFE 40%	34.27	5.57	−5.70	1.23	38.67

**FIGURE 4:**
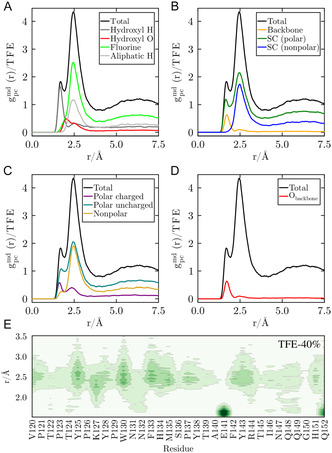
Total MDDF of TFE at 40% (v/v) in black, and decomposition into atoms or atom groups of (A) TFE, (B) backbone, and side chains (polar and nonpolar) of the peptide, (C) polar (charged and uncharged) and nonpolar residues of the protein, and (D) backbone‐carbonyl oxygen. (E) 2D density map per residue in the vicinity of the S2 region of the Esg protein.

The MDDFs can be decomposed into chemical group contributions, providing a molecular interpretation of solute–solvent interactions [47, 49]. In essence, the decomposition of MDDFs displays the frequency with which each atom (or group of atoms) is the closest to each solute atom at each distance, with the sum of all contribution curves equaling the total MDDF. Figure 4 shows the MDDF of the TFE at 40% (v/v) decomposed as a function of atoms and group atoms of the TFE (Figure 4A) and the protein (Figure 4B–D). Figure 4A shows that the first TFE peak (green curve), related to direct hydrogen bonds between TFE and protein atoms, is completely determined by the contribution of the hydroxyl hydrogen. TFE interacts by hydrogen bonds with the polar residues of the side chains (Figure 4B,C) and with the backbone (essentially, with the carbonyl oxygen of the backbone (Figure 4D). The direct interactions of TFE with the backbone have a destabilizing effect on the helical structure, as recently reported [24].

Nonspecific interactions are established, mainly through interactions involving fluorine and aliphatic hydrogens (Figure 4A), which interact with the side chains of the protein, mainly with polar uncharged and nonpolar amino acids (Figure 4C).

Figure 4E depicts the map of TFE density distribution in a system containing a 40% (v/v) TFE. The darker the color, the greater the TFE density relative to the bulk density of TFE. The examination of the protein surroundings (between 1.5 and 3.5 Å) shows stronger TFE interactions with valine (V120), tyrosine (Y125, Y138, and Y143), lysine (K127), histidine (H134 and H151), arginine (R144), tryptophan (W130), phenylalanine (F133 and F142), methionine (M135), and proline (P137) residues. E141 is the anionic amino acid that most frequently interacts with TFE through hydrogen bonds. Interestingly, E141 also interacts with water and forms a salt bridge with R144 (Figure S1). In TFE‐40%, this salt bridge is more stable than in pure water. Thus, the TFE competition with water for hydrogen bonding with E141 (Figure S2) favors the intramolecular interaction with R144 contributing to helix stabilization, as previously suggested [13].

## 4 Conclusions

In this work, we explored the conformational landscape of the S2 region of the Esg protein in the presence of TFE using CD spectroscopy and enhanced sampling molecular dynamics simulations. This region has previously been associated with functions that complement the transcriptional repressor activity of the C‐terminal domain when it adopts an ordered conformation. Our results show that residues H134 to G150 exhibit a strong propensity to adopt an *α*‐helical conformation, with the maximum *α*‐helix content observed at TFE‐40% (v/v). The cosolvent and peptide interact primarily through nonspecific interactions between fluorine atoms and aliphatic hydrogens of TFE, with polar uncharged and neutral regions of the peptide being predominant. Additionally, direct interactions also occur and are dominated by the hydroxyl hydrogen of the TFE and the side chains and backbone of the peptide. These interactions collectively drive the preferential solvation of the peptide by TFE. Overall, these findings support the view that the S2 region may play a structural role in modulating Esg function and provide a basis for future studies investigating its interactions with nucleic acids or other regulatory partners.

## Supporting Information

Additional supporting information can be found online in the Supporting Information section. The Supporting Information file includes tables (Tables S1 and S2) and figures (Figures S1–S4) with additional analyses. Table S1 reports the solvent‐accessible surface area (SASA) for the S2 region of the Esg protein, and Table S2 summarizes the average number of hydrogen bonds (HBs). Figure S1 shows the number of hydrogen bonds (HBs) formed by the Glu141 residue as a function of time. Figure S2 highlights the 2D density map per residue of the water. Figure S3 presents the average *α*‐helix content over time, and Figure S4 displays the corresponding probability distributions of helical content. Finally, Figure S5 provides the convergence analysis of the simulations. **Supporting** **Fig.** **S1:** Number of hydrogen bonds (HBs) as a function of time formed by the Glu141 residue. In pure water, HBs are shown between Glu141 and (A) Arg144 (0.37 ± 0.02) and (B) water molecules (5.12 ± 0.03). In the TFE/water mixture, HBs are shown between Glu141 and (C) Arg144 (0.43 ± 0.02), (D) water molecules (2.36 ± 0.04), and (E) TFE molecules (2.12 ± 0.04). Values represent the mean number of HBs ± standard deviation. **Supporting** **Fig.** **S2:** 2D density map per residue of the water in the vicinity of the S2 region of the Esg protein at 40% (v/v) TFE solution. **Supporting** **Fig. S3:** Average *α*‐helix content as a function of replica exchange time for the S2 region of Esg protein in (A) water (blue line) and in TFE solutions: (B) 10% (orange line), (C) 40% (green line), and (D) 100% (red line) of TFE. **Supporting** **Fig.** **S4:** Distributions of the probability of finding helical content for the S2 region of the Esg protein in all simulations. In any TFE solution, the increase in ellipticity is associated with a lower probability of the protein having an *α*‐helix content equal to zero. **Supporting** **Fig.** **S5:** Convergence of the simulations and the statistical error of the average *α*‐helical content. Statistical analysis was assessed using block averaging analysis for each system. **Supporting Table S1:** Solvent Accessible Surface Area (SASA) for the S2 region of the Esg protein. **Supporting Table S2:** Average number of Hydrogen Bonds (HBs) between protein (P), water (W), and 2,2,2‐Trifluoroethanol (TFE).

## Funding

This study was supported by the Centros de Pesquisa, Inovação e Difusão, Fundação Amazônia Paraense de Amparo à Pesquisa (2013/08293‐7), Fundação de Amparo à Pesquisa do Estado de São Paulo (2019/17874‐0, 2025/03933‐5, 2025/03946‐0), and Coordenação de Aperfeiçoamento de Pessoal de Nível Superior (301909/2022‐9, 206‐04/092018).

## Conflicts of Interest

The authors declare no conflicts of interest.

## Supporting information

Supplementary Material

## Data Availability

The data that support the findings of this study are available in the supplementary material of this article.
