# Supplementary material for: Preferential Solvation by Trifluoroethanol Drives α‐Helical Folding in the Disordered S2 Region of the Escargot Protein
Source: Chemphyschem. 2026 Feb 10;27(3):e202500668. doi: 10.1002/cphc.202500668 (PMC12890072; doi:10.1002/cphc.202500668)
Supplement: Supplementary file 1 — Supplementary Material [file CPHC-27-e202500668-s001.pdf]

Supporting Information

# Preferential Solvation by Trifluoroethanol Drives $\alpha$-Helical Folding in the Disordered S2 Region of the Escargot Protein

*Vinicius Piccoli[1,*], Ander F. Pereira[1,*], Lina Rivillas-Acevedo[2], Nina Pastor[2] , Ángel E. Peláez-Aguilar[3], and Leandro Martínez[1,‡]*

[1]Institute of Chemistry and Center for Computing in Engineering & Science, University of Campinas, Campinas, SP, Brazil

[2]*Centro de Investigación en Dinámica Celular, Universidad Autónoma del Estado de Morelos, México.*

[3] *Departamento de Microbiología Molecular, Instituto de Biotecnología, Universidad Nacional Autónoma de México, Cuernavaca, Morelos, México*

*These authors contributed equally to this work. ‡To whom correspondence should be addressed.

**Contents**

**Table S1:** Solvent Accessible Surface Area (SASA) for the S2 region of the Esg protein — Page 2

**Table S2:** Average number of Hydrogen Bonds (HBs) — Page 2

**Figure S1:** Number of hydrogen bonds (HBs) as a function of time formed by the Glu141 residue — Page 3

**Figure S2:** 2D density map per residue of the water — Page 4

**Figure S3:** Average $\alpha$-helix content as a function of time — Page 5

**Figure S4:** Distributions of the probability of finding helical content — Page 6

**Figure S5:** Convergence analysis of the simulations — Page 8

**Section 1: Structural properties of the S2 region of the Esg protein**

**Table S1**. Solvent Accessible Surface Area (SASA) for the S2 region of the Esg protein.

| Systems | Solvent Accessible Surface Area (SASA) ($nm^2$) |
|---|---|
| S2 region - 0% v/v | 31.25 ± 0.06 |
| S2 region - 10% v/v | 32.68 ± 0.06 |
| S2 region - 40% v/v | 34.28 ± 0.08 |
| S2 region - 100% v/v | 35.12 ± 0.07 |

**Table S2**. Average number of Hydrogen Bonds (HBs) between protein (P), water (W), and 2,2,2-Trifluoroethanol (TFE).

| Systems | Average number of HBs | |
|---|---|---|
| | (P-W) | (P-TFE) |
| S2 region - 0% v/v | 83.0 ± 0.2 | - |
| S2 region - 10% v/v | 74.5 ± 0.2 | 3.39 ± 0.06 |
| S2 region - 40% v/v | 53.3 ± 0.2 | 12.5 ± 0.1 |
| S2 region - 100% v/v | - | 42.1 ± 0.1 |

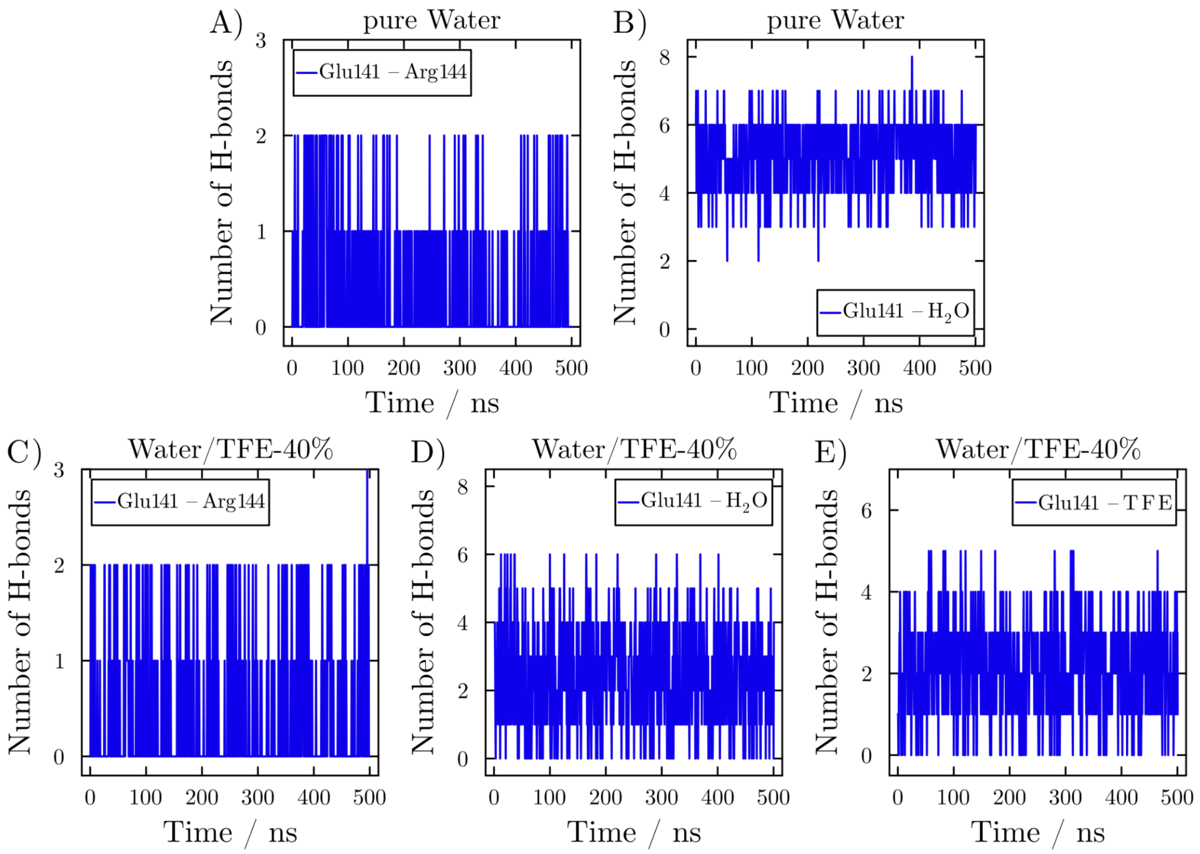


**Figure S1**. Number of hydrogen bonds (HBs) as a function of time formed by the Glu141 residue. In pure water, HBs are shown between Glu141 and A) Arg144 (0.37 ± 0.02) and B) water molecules (5.12 ± 0.03). In the TFE/water mixture, HBs are shown between Glu141 and C) Arg144 (0.43 ± 0.02), D) water molecules (2.36 ± 0.04), and E) TFE molecules (2.12 ± 0.04). Values represent the mean number of HBs ± standard deviation.

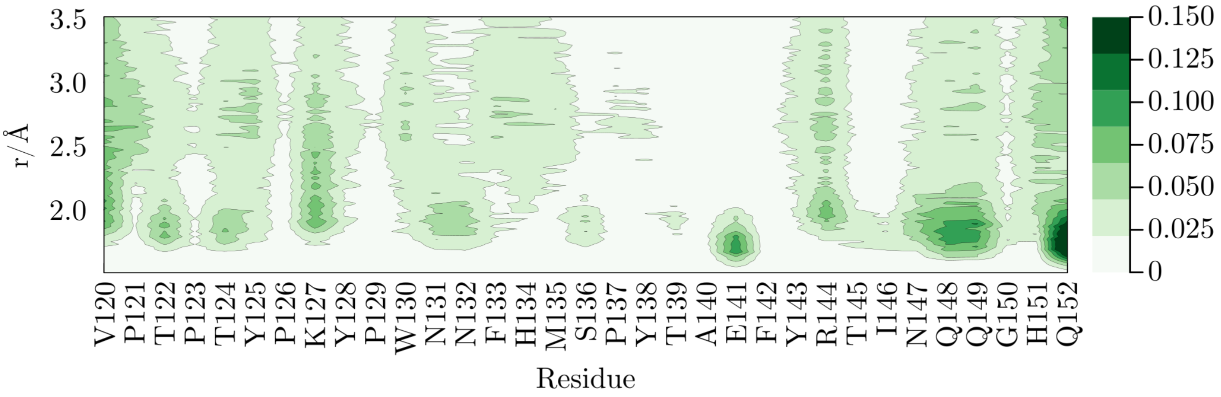


**Figura S2**. 2D density map per residue of the water in the vicinity of the S2 region of the Esg protein at 40% (v/v) TFE solution.

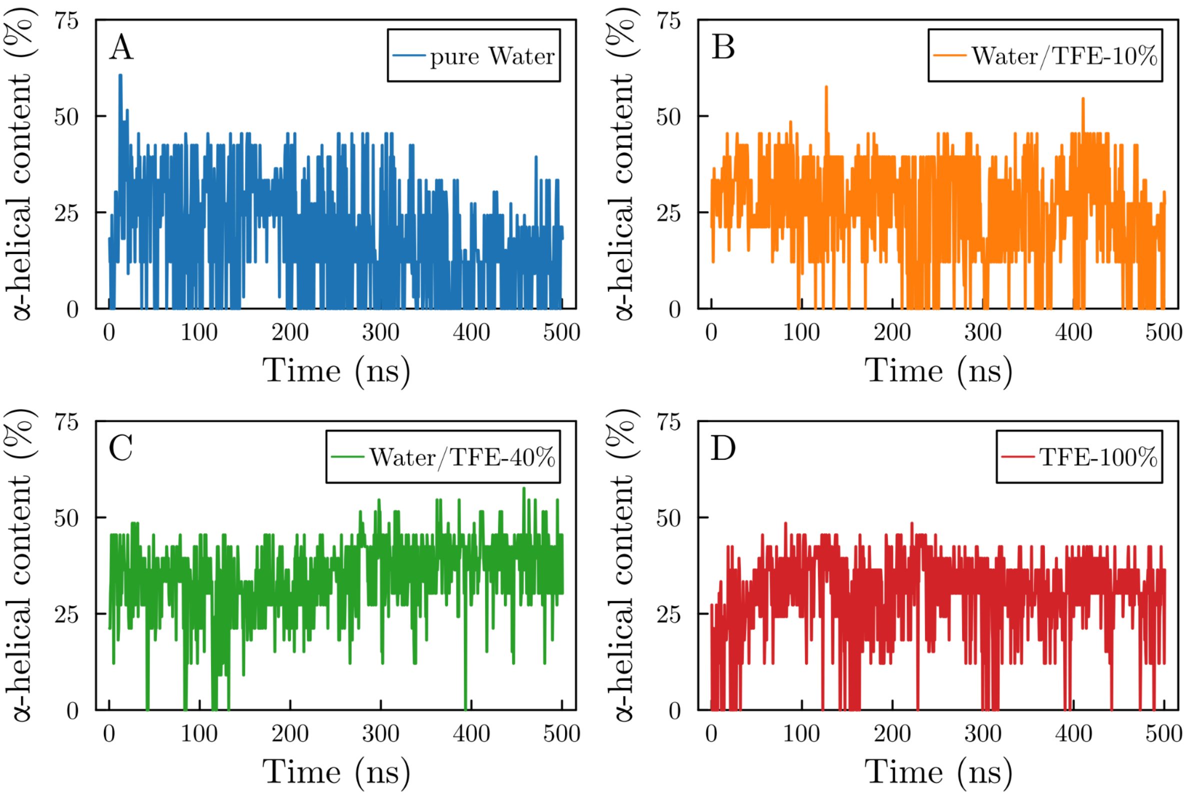


**Figure S3**. Average $\alpha$-helix content as a function of Replica-Exchange time for the S2 region of Esg protein in A) water (blue line) and in TFE solutions: B) 10% (orange line), C) 40% (green line), and D) 100% (red line) of TFE.

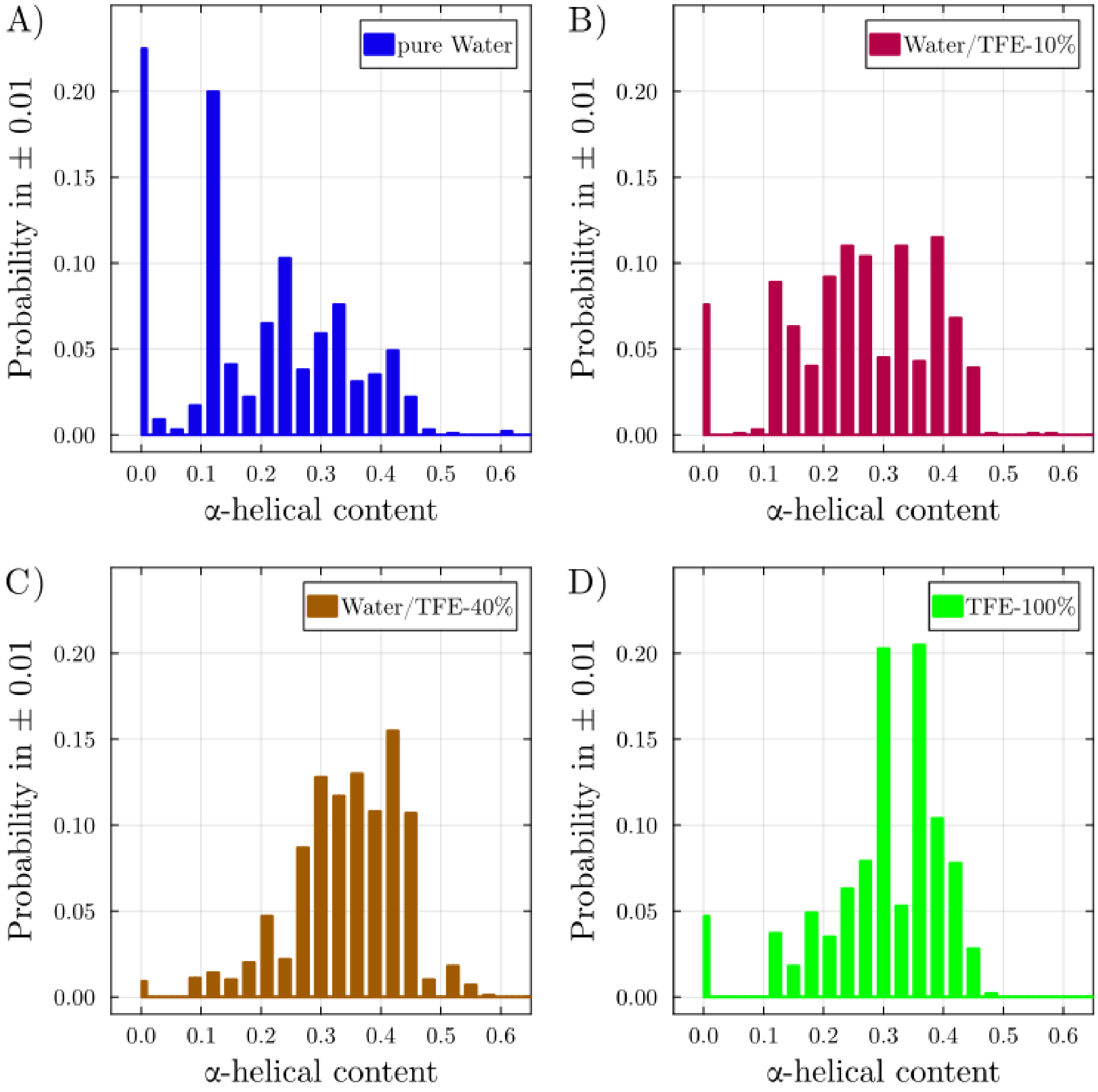


**Figure S4**. Distributions of the probability of finding helical content for the S2 region of the Esg protein in all simulations. In any TFE solution, the increase in ellipticity is associated with a lower probability of the protein having an $\alpha$-helix content equal to zero.

**Section 2: Convergence analysis of the simulations**

The plots below show the convergence of the mean, the standard error estimate as a function of block size, and the autocorrelation function of the time series data. Distributions of the probability of finding helical content for the S2 region of the Esg protein in all simulations. In any TFE solution, the increase in ellipticity is associated with a lower probability of the protein having an $\alpha$-helix content equal to zero. For all systems, the stabilization of the mean value with increasing block size confirms that the simulation lengths were sufficient to obtain statistically reliable averages for the $\alpha$-helical content.

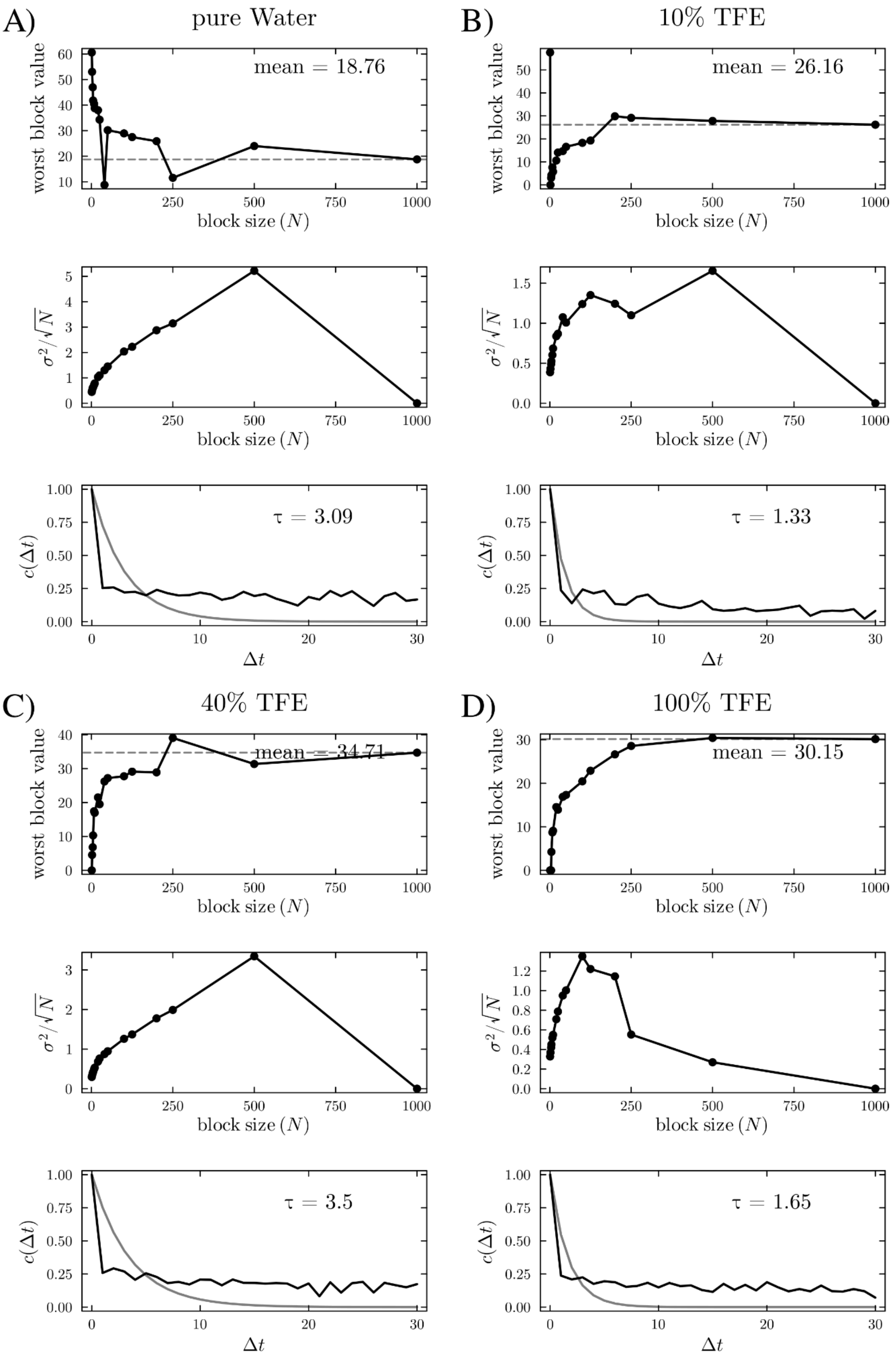


**Figure S5**. Convergence of the simulations and the statistical error of the average $\alpha$-helical content. Statistical analysis was assessed using block averaging analysis for each system.
